# Waste Control by Waste: Red Mud-Based Porous Carbothermal Composite for Efficient Remediation of Manganese and Ammonia Nitrogen in Contaminated Soil

**DOI:** 10.3390/ma19143076

**Published:** 2026-07-17

**Authors:** Xinyue Shi, He Shang, Lei Wang, Hongxia Li, Meilin Liu, Yingchun Sun

**Affiliations:** 1 National Engineering Research Center for Environment-Friendly Metallurgy in Producing Premium Non-Ferrous Metals, China GRINM Group Co., Ltd., Beijing 101407, China; shixinyue@grinm.com (X.S.); shanghe@grinm.com (H.S.); lihongxia@grinm.com (H.L.); liumeilin@grinm.com (M.L.); 15966001661@163.com (Y.S.); 2 GRINM Resources and Environment Tech. Co., Ltd., Beijing 101407, China; 3 Beijing Engineering Research Center of Strategic Nonferrous Metals Green Manufacturing Technology, China GRINM Group Co., Ltd., Beijing 101407, China; 4 General Research Institute for Nonferrous Metals, Beijing 100088, China

**Keywords:** manganese, ammonia nitrogen, solid waste resource utilization, manganese slag-contaminated soil

## Abstract

The co-contamination of manganese ions (Mn^2+^) and ammonia nitrogen (NH_4_^+^) caused by the stockpiling of manganese residue poses a serious threat to the ecological environment. In this study, a series of the composite was prepared from red mud, bentonite, and corn straw via oxygen-limited pyrolysis. The effects of pyrolysis temperature and raw material ratio on the material properties were investigated, and the synergistic remediation performance of the composites for Mn^2+^ and NH_4_^+^ in manganese residue-contaminated soil was evaluated through a 180-day soil column experiment. The results showed that the composite prepared with a raw material ratio of 1:1:1 at a pyrolysis temperature of 700 °C exhibited the largest specific surface area and the most developed pore structure, achieving a Mn^2+^ removal rate of 92.72% ± 0.85% in aqueous solution. In the soil column experiment, the material prepared at 700 °C gave the highest immobilization rate for soil Mn^2+^ (96.22% ± 0.5%), whereas the combined addition of materials prepared at 700 °C and 500 °C achieved the best removal efficiency for NH_4_^+^ (99.33% ± 0.23%). Mechanistic studies revealed that the stabilization of Mn^2+^ is primarily attributable to alkaline precipitation and mineral lattice solid solution induced by the composite, leading to the formation of stable spinel phases (e.g., (Fe,Mn)_3_O_4_) and insoluble manganese phosphate-carbonate salts. The removal of NH_4_^+^ is proposed to proceed via adsorptive enrichment by the porous structure and Fe^0^-mediated Fenton-like catalytic oxidation, ultimately converting NH_4_^+^ to N_2_ gas. The 180-day monitoring results demonstrated that the remediation effect continuously increased over time, indicating good long-term stability of the composite. This study provides an efficient, low-cost functional material derived from solid waste for the remediation of manganese residue-contaminated soil and offers a theoretical basis for the synergistic resource utilization of multiple solid wastes.

## 1. Introduction

Manganese slag is a typical large-scale industrial solid waste produced in the electrolytic manganese industry. Its storage not only occupies a large amount of land, but also poses a serious environmental hazard due to the presence of high concentrations of soluble manganese ions (Mn^2+^) and ammonia nitrogen (NH_4_^+^) [1]. Under the influence of rainwater leaching, Mn^2+^ is prone to migrate into water bodies and soil, posing threats to the ecosystem and human health [2,3]. Furthermore, the release of NH_4_^+^ can lead to water eutrophication, further intensifying the environmental risks [4]. For the remediation of manganese slag-contaminated soil, traditional physical and chemical methods such as soil replacement and chemical leaching are often costly, prone to damaging soil structure, and may even pose a risk of secondary pollution [5,6]. Therefore, developing an economic, efficient, and environmentally friendly in situ remediation technology to achieve the simultaneous stabilization/removal of Mn^2+^ and NH_4_^+^ is of great practical significance.

In recent years, the collaborative preparation of functional materials using industrial solid waste and agricultural and forestry waste has become a research hotspot in the field of environmental remediation [7,8]. Among them, red mud, as a strong alkaline industrial waste residue rich in iron and aluminum, can efficiently capture heavy metal ions through adsorption and co-precipitation due to its unique alkaline environment, porous structure, and high-activity iron oxides [9]. Bentonite, with its extremely large specific surface area, excellent cation exchange capacity (CEC), and water absorption and expansion properties, is widely used to enhance the mechanical strength of materials and its adsorption capacity for heavy metals [10,11]. Furthermore, carbon-rich agricultural and forestry biomass can be pyrolyzed under oxygen-limited conditions to produce biochar [12]. This product has a well-developed pore structure, a large specific surface area, abundant surface functional groups, and excellent adsorption properties [13]. It is widely used in environmental remediation, soil improvement, and carbon sequestration and emission reduction, among other fields [14,15]. Based on this, co-pyrolyzing these three substances is expected to construct a red mud-based porous carbon thermal composite material integrating adsorption, alkaline precipitation, and catalytic oxidation, achieving the goal of “waste treatment by waste”.

Although previous studies have separately investigated the remediation performance of red mud and biochar for individual pollutants [16,17,18], the synergistic remediation mechanism for manganese slag composite pollution (Mn^2+^ and NH_4_^+^) remains unclear. In particular, the phase evolution rules of the three components under different ratios and pyrolysis temperatures, the regulation mechanism of microstructure, and how the redox process in the material couples with the alkaline environment to achieve the immobilization of Mn^2+^ and the removal of NH_4_^+^ have not yet been systematically studied. Based on this, this study used red mud, bentonite, and corn straw as raw materials to prepare a series of carbon-based composite materials by regulating the pyrolysis temperature (300–700 °C) and the ratio of raw materials. By integrating modern analytical techniques (SEM-EDS, XRD, XPS, BET) and soil incubation experiments, the aim is to: (1) reveal the influence laws of pyrolysis conditions on the microstructure and phase composition of materials; (2) evaluate the differences in the transformation of Mn^2+^ immobilization forms and the removal efficiency of NH_4_^+^ in soil among different materials; (3) clarify the cooperative removal mechanism of Mn^2+^ and NH_4_^+^ at the material interface. The research results aim to provide an efficient solid waste-based material for the remediation of manganese slag-contaminated soil, and to offer a theoretical basis for the collaborative resource utilization of multiple solid wastes.

## 2. Materials and Methods

### 2.1. Preparation of Red Mud-Based Porous Carbon Thermal Composite Material

The raw materials of the composite mainly include red mud, bentonite, and corn straw biomass. Among them, the red mud was obtained from an alumina production company in Guangxi, the pH value of the red mud is 9.5, and its elemental and mineral compositions are shown in Table S1 and Figure S1a, respectively. The bentonite was purchased from a mineral product processing factory in Hebei, the cation exchange capacity of the bentonite is 125 mmol/100 g, and its elemental and mineral compositions are shown in Table S2 and Figure S1b, respectively. The corn straw biomass was bought from a biomass processing company in Shandong. The red mud was dried, crushed, and sieved through a 120-mesh sieve to obtain red mud powder. Bentonite was dried and then sieved through a 120-mesh sieve to get bentonite powder. Corn straw was naturally dried, crushed, washed, vacuum-dried, and then sieved through a 120-mesh sieve to obtain straw powder. The pre-treated red mud and bentonite powder were added to 250 mL of deionized water in a mass ratio of 1:1, then stirred with a magnetic stirrer for 2 h to obtain a stable suspension of red mud and bentonite. The corn straw biomass and the prepared suspension were placed in a 300 mL wide-mouth conical flask and shaken in a 200 rpm shaker for 24 h to ensure that the biomass material and the suspension were fully mixed, forming a ternary mixed slurry. After the obtained slurry was dried in an oven for 3 days, it was transferred to a filter paper and further dried for 1 day to obtain the precursor of the composite material. The precursor was placed in a tube furnace and sintered for 2 h at 300, 400, 500, 600, and 700 °C, respectively, in a nitrogen atmosphere, resulting in different temperature conditions of red mud-based porous carbon-thermal composite materials. To investigate the effects of different ratios, the red mud, bentonite, and corn straw were mixed in mass ratios of 1:1:1, 1:1:3, 1:1:5, and 1:1:10 to prepare the composite materials under different conditions. The obtained composite materials were ground and passed through a 100-mesh sieve for subsequent use.

### 2.2. Characterization of Red Mud-Based Porous Carbon Composite Materials

In order to better understand the properties of the red mud-based porous carbon thermal composite material, the contents of C, H, O, and N were detected using an elemental analyzer (UNICUBE, Elementar Analysensysteme GmbH, Langenselbold, Germany) by direct combustion method, with oxygen content determined directly by the instrument’s oxygen detection module. The specific surface areas of the materials were measured by the BET/N_2_-adsorption method (BET, ASAP 2460, Micromeritics Instrument Corporation, Norcross, GA, USA). The morphology and microstructure of the materials were observed using scanning electron microscopy and X-ray energy spectroscopy (SEM-EDX, Quanta FEG250, FEI Company, Hillsboro, OR, USA). Additionally, the active functional groups were examined using MIR spectroscopy (FTIR, Nicolet iS5, Thermo Fisher Scientific, Waltham, MA, USA). The elements present in the material and their oxidation states were analyzed using X-ray photoelectron spectroscopy (XPS) (XPS, Axis Ultra, Kratos Analytical Ltd., Manchester, UK).

### 2.3. Sequencing Batch Experiment

A series of experiments were conducted to explore the optimal process parameters of the composite. The mass ratio of red mud, bentonite, and corn straw was set at 1:1:1, and the pyrolysis temperatures were set at 300 °C, 400 °C, 500 °C, 600 °C, and 700 °C, respectively, to explore the influence of preparation temperature on the material properties. The pyrolysis temperature was fixed at 700 °C, and the mass ratios of red mud, bentonite, and corn straw are set as 1:1:1, 1:1:3, 1:1:5, and 1:1:10, respectively, to investigate the influence of the mass ratios of biomass on the material properties. The composite prepared under different conditions were, respectively, added to the mixed solution with a concentration of 40 mg/L for Mn^2+^ and 10 mg/L for NH_4_^+^. The samples were shaken in a shaker at 150 rpm, 25 °C for 24 h. The initial pH of the solution was 6.8 ± 0.2. Then they were taken out, filtered through a 0.45 μm filter membrane, and the concentrations of manganese ions and ammonia nitrogen ions in the original solution and the solution after the composite materials adsorption were determined by ICP-OES. The removal efficiency of manganese and ammonia nitrogen in the solution by the composite materials was calculated.

All experiments were conducted in three independent replicates. Data were expressed as mean ± standard deviation (SD).

### 2.4. Long-Term Experimental Procedure

To explore the remediation effect of the composite on manganese and ammonia nitrogen in manganese slag-contaminated soil, a soil column experiment was conducted. The leaching concentrations of manganese and ammonia nitrogen in the soil contaminated by manganese slag were 17.9 mg/L and 11.8 mg/L, respectively. One blank control group and three experimental groups were set up in the experiment. In experimental group 1, 3% of the red mud-based porous carbon thermal composite material prepared at 700 °C was added; in experimental group 2, 3% of the composite material prepared at 500 °C was added; in experimental group 3, 1.5% of the composite material prepared at 700 °C and 1.5% of the composite material prepared at 500 °C were added, respectively. The 3% addition dose was selected based on preliminary screening experiments (1%, 3%, 5%, and 10% by weight), which showed that 3% achieved an optimal balance between remediation performance and material economy. This dosage is also consistent with commonly reported amendment rates in soil remediation studies [19]. The experiment was continuously monitored for 180 days. The manganese ions and ammonia nitrogen ions in the soil were extracted using the HJ/T 299-2007 [20], and the concentrations of Mn^2+^ and NH_4_^+^ ions were determined by ICP-OES. The solution pH and ORP were determined by HI 3221 pH/ORP meter (HI 3221, Hanna Instruments Inc., Woonsocket, RI, USA). The Tessier five-step sequential extraction method was adopted to determine the speciation of manganese ions in the soil before and after remediation. The composition of the reaction products was detected and analyzed by powder X-ray diffraction (XRD, Ultima IV, Rigaku Corporation, Tokyo, Japan).

## 3. Results and Discussion

### 3.1. Characterization of Composite Materials

#### 3.1.1. Surface Elemental Composition

Elements are the fundamental building blocks of the chemical structure of carbon-based materials, with C, H, O, and N constituting the main framework of such materials [21]. By analyzing the elemental proportions in carbon-based materials, the properties of materials prepared under different conditions can be inferred [22]. For example, the H/C and O/C molar ratios are important parameters for characterizing the degree of carbonization and aromaticity of carbon-based materials, while the (O + N)/C ratio serves as a polarity index [23]. The elemental compositions of the composites prepared at different temperatures (300–700 °C) with a fixed raw material ratio (red mud:bentonite:corn straw = 1:1:1), as well as those prepared at 700 °C with various ratios (1:1:1, 1:1:3, 1:1:5, 1:1:10), are presented in Table 1. The elemental composition of the corn straw biochar is presented in Table S3. As shown in the table, with increasing pyrolysis temperature, the H/C and O/C molar ratios continuously decrease, indicating enhanced aromaticity and a higher degree of carbonization of the composites. Meanwhile, the (O + N)/C molar ratio also decreases, suggesting a reduction in surface polar functional groups. At the same temperature, as the biomass proportion increases, the C content gradually rises, whereas the H/C and O/C molar ratios show little variation, indicating that the degree of carbonization is primarily governed by the pyrolysis temperature rather than by the material ratio.

#### 3.1.2. Specific Surface Area

The specific surface area is a quantitative indicator of the degree of pore development and particle fineness of a material, and it affects the physical and chemical properties of the material [24]. The pore structure of the composites prepared at different temperatures (300–700 °C) with a fixed raw material ratio (red mud:bentonite:corn straw = 1:1:1), as well as those prepared at 700 °C with various ratios (1:1:1, 1:1:3, 1:1:5, 1:1:10), are presented in Table 2. The results indicate that the specific surface area of all the composites is significantly higher than that of the pure biochar analyzed previously [25]. This suggests that the addition of red mud and bentonite has a significant synergistic effect with corn straw, greatly promoting the development of the pore structure of the composites. When the raw material ratio is fixed at 1:1:1, the specific surface area continuously and significantly increases with the rise of pyrolysis temperature. This might be due to the fact that high temperature promotes the deep carbonization of corn straw, allowing more volatile matter to be released fully and forming abundant pores. At the final pyrolysis temperature of 700 °C, by changing the proportion of corn straw, the specific surface area of the material also changed. When the mass ratio of red mud, bentonite and corn straw biomass was 1:1:1, the specific surface area of the composite material was the largest. This was because the inorganic components of red mud and bentonite formed an ideal “composite framework” with the produced biochar. Control experiments with pure corn straw biochar (without red mud or bentonite) showed a significantly lower specific surface area (28.5 m^2^/g) and a collapsed pore structure, confirming that the inorganic components of red mud and bentonite act as rigid templates to prevent pore collapse during pyrolysis [26,27]. Therefore, within the scope of this study, when the ratio is 1:1:1 and the pyrolysis temperature is 700 °C, the composite material with the most developed pore structure can be obtained.

#### 3.1.3. Microscopic Morphology Analysis

**Figure 1:**
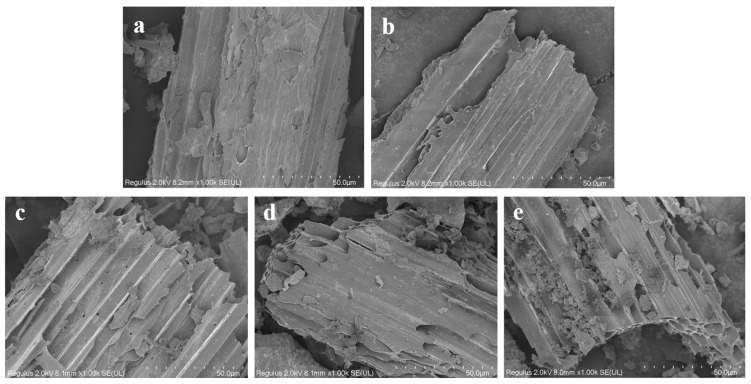
SEM images of the composite materials at different temperatures. (**a**) 1:1:1-300 (**b**) 1:1:1-400 (**c**) 1:1:1-500 (**d**) 1:1:1-600 (**e**) 1:1:1-700.

The SEM images of the ternary composite materials prepared at different temperatures (300–700 °C) with the ratio of red mud, bentonite, and corn straw being 1:1:1 are shown in Figure 1. At 300 °C, the red mud, bentonite, and straw fibers are mainly in a physically mixed state, resulting in a relatively low specific surface area of only 28.38 m^2^/g. At 400 °C, the straw fibers begin to contract and deform, and cracks appear on the surface; some of the red mud particles are partially embedded in the carbon layer, resulting in a small number of micropores (1–2 μm), but the overall process is still dominated by physical mixing. At 500 °C, the cellulose in the straw undergoes intense decomposition, forming a honeycomb-like porous carbon framework. The iron and manganese oxides in the red mud are reduced by carbon to generate nano-zero-valent iron particles (bright white spots), and the density of mesopores (2–50 nm) significantly increases. At 600 °C, the carbon framework further undergoes graphitization, and the pore walls become thinner. The Na/Al silicate in the red mud melts to form a glass phase (the gray smooth area), which partially blocks the pores but enhances the interfacial bonding force. At 700 °C, a large amount of ash melts and forms a continuous enamel layer, covering part of the carbon framework; the remaining pores are mainly isolated large pores, and the microporosity decreases, causing the material to tend towards densification. Notably, although the molten enamel layer partially covers the carbon surface at 700 °C (Figure 1e), the internal mesoporous structure remains well developed, as evidenced by the BET results (Table 2). This indicates that the surface morphology observed by SEM does not fully represent the overall porosity, and the internal pores generated by volatile release and stabilized by the inorganic template contribute predominantly to the high specific surface area.

**Figure 2:**
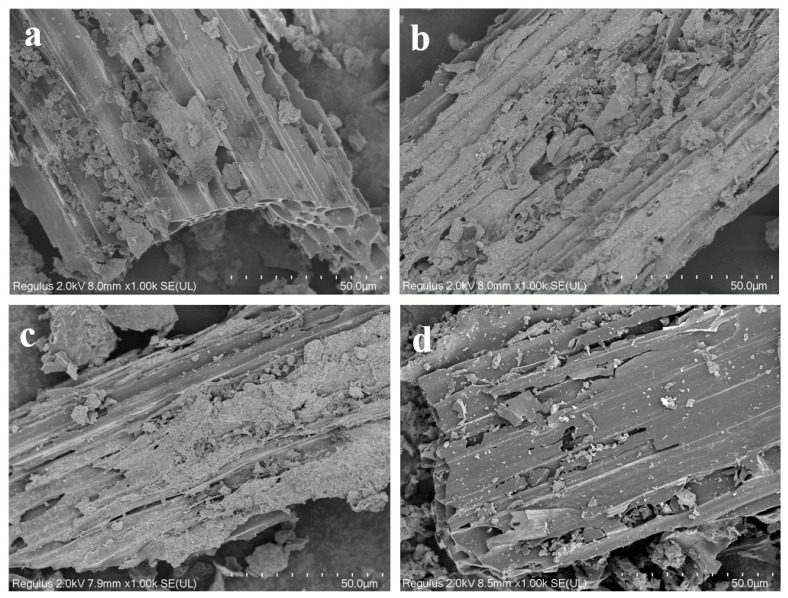
SEM images of the composite materials at different ratios (**a**) 1:1:1-700 (**b**) 1:1:3-700 (**c**) 1:1:5-700 (**d**) 1:1:10-700.

Besides the pyrolysis temperature, the composition ratio of the composite is also critical. As shown in Figure 2, the results indicate that when the ratio is 1:1:1, the formed composite material has a continuous enamel layer on its surface. The red mud–bentonite molten phase covers most of the carbon framework, and at the same time, a micrometer-scale crack network is distributed. This structure can not only physically encapsulate pollutants but also provide ion diffusion channels. Under the 1:1:3 ratio, the excessive development of the carbon framework dilutes the active sites of the minerals, resulting in a decrease in catalytic ability. At the 1:1:5 and 1:1:10 ratios, the higher biomass content leads to a relative reduction in the mineral-derived active components (Fe_2_O_3_, Al_2_O_3_), which may contribute to the decreased removal efficiencies.

#### 3.1.4. Functional Group Analysis

**Figure 3:**
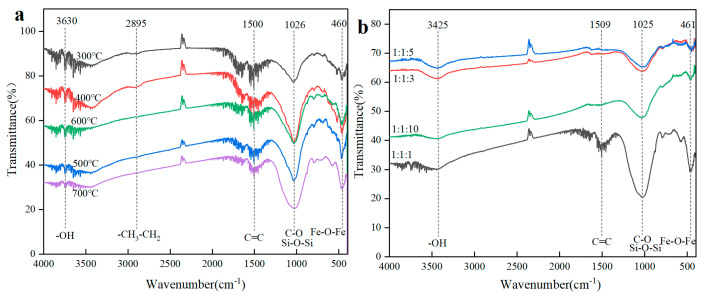
FTIR spectrum (**a**) composite materials prepared at different decomposition temperatures (**b**) composite materials prepared at different ratios.

FTIR (Fourier transform infrared) spectroscopy has been widely used to characterize the functional groups on the surface of carbon-based materials. The FTIR spectra of functional groups in the composites produced at different temperatures are distinct (Figure 3a). The absorption peak at 3630 cm^−1^ corresponds to the surface lattice hydroxyl groups [28]. As the temperature increases, the intensity significantly decreases, indicating that thermal-induced dehydration and hydroxyl condensation occur. The weak peak at 2895 cm^−1^ corresponds to the C–H stretching vibration [28]. Its intensity decreases with the increase in temperature, indicating the carbonization or thermal decomposition of the carbon skeleton. The broad peak around 1500 cm^−1^ is the superposition of C=O and the C=C of the aromatic ring [29]. The intensity has slightly decreased but the peak position has not significantly shifted, indicating that the oxygen-containing functional groups are continuously removed and the degree of carbonization and aromatization is increasing [28]. The carbonization and aromatization trends are further confirmed by the elemental analysis (Table 1), where the H/C ratio drops from 0.15 at 300 °C to 0.03 at 700 °C, indicating enhanced aromaticity [30]. The decreasing O/C ratio also reflects the loss of oxygen-containing groups, consistent with the FTIR results (Figure 3a); 1026 cm^−1^ corresponds to the C–O stretching vibration [31]. The intensity decreases with the increase in temperature, indicating that the interaction between the carbon layer and the metal oxide interface weakens. The Fe–O–Fe lattice vibration peak at 460 cm^−1^ becomes sharper and more intense as the temperature rises, indicating an increase in the orderliness of the oxide lattice and grain growth [32].

Figure 3b shows the FTIR spectra of the composite materials prepared at 700 °C with different raw material ratios (1:1:1, 1:1:3, 1:1:5, 1:1:10). The broad peak at 3425 cm^−1^ corresponds to –OH, and its intensity significantly increases with the increase in the proportion of biomass. The absorption peak at 1509 cm^−1^ (C=O and aromatic C=C) also intensifies with the increase in the proportion of biomass, indicating an increase in the proportion of carbonaceous components and an increase in the total amount of oxygen-containing functional groups. The strong absorption band at 1025 cm^−1^ is attributed to the superposition of Si-O-Si and Si-O-Al stretching vibrations from the bentonite framework, along with C-O-C/C–OH from the biomass-derived carbon. The intensity of this band decreases with increasing biomass ratio (Figure 3b), consistent with the dilution of the bentonite component. The intensity of the 461 cm^−1^ peak gradually decreased as the proportion of red mud decreased, which was consistent with the change in the raw material ratio.

#### 3.1.5. XPS Analysis

**Figure 4:**
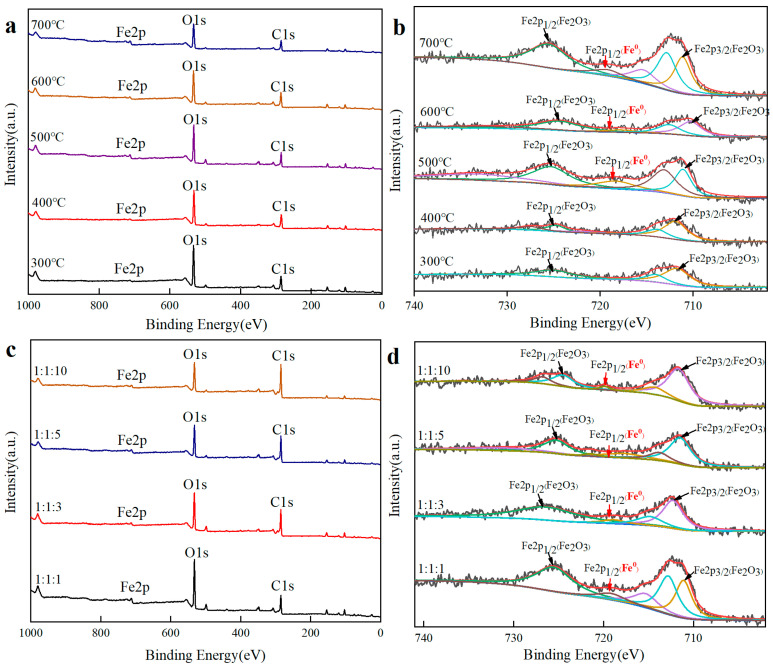
XPS Spectrum (**a**) The XPS total spectra of the composite materials prepared at different decomposition temperatures (**b**) The Fe spectra of the composite materials prepared at different decomposition temperatures (**c**) The XPS total spectra of the composite materials prepared at different ratios (**d**) The Fe spectra of the composite materials prepared at different ratios.

To analyze the elemental composition and valence state of the composite materials, XPS tests were conducted on the samples under different decomposition temperatures and ratios. Figure 4a shows that characteristic peaks were observed at 712 eV (Fe2p), 532 eV (O1s), and 285 eV (C1s) for all samples. As the temperature increased, the Fe2p peak became stronger (the strongest at 700 °C), while the C1s and O1s peaks weakened, which was attributed to the escape of carbon oxidation and the decomposition of oxygen-containing functional groups. Figure 4b shows that at temperatures ranging from 300 to 400 °C, Fe exists only as Fe^3+^ (Fe_2_O_3_). At 500 °C, a peak at ~720 eV, which is consistent with the binding energy of Fe^0^, is observed in the XPS spectra. This suggests that partial reduction of Fe^3+^ to Fe^0^ by carbon may occur at elevated temperatures. It should be noted that Fe^0^ is an intermediate species formed during the carbothermal reduction process and is readily oxidized upon exposure to air. Therefore, its detection and quantification are inherently challenging. In this study, the formation of Fe^0^ in the 500 °C composite is suggested by SEM-EDS. However, the results should be interpreted with caution, as quantitative determination was not performed. In Figure 4c, the C1s peak significantly intensifies as the biomass proportion increases, confirming that the biomass carbonization forms a carbon framework. The relative intensity of the O1s peak decreases as the proportion increases, and the intensity of the Fe2p peak decreases as the proportion increases, due to the dilution of the carbon signal relative to the iron signal. In Figure 4d, the Fe^3+^ signal decreases as the proportion increases, and the Fe^0^ peak has the highest intensity in the 1:1:1 sample. Therefore, for reactions that require the use of iron catalytic activity, a low biomass proportion formulation should be adopted to ensure the presence of active iron sites on the surface.

### 3.2. Removal Performance of Different Composite Materials for Mn^2+^ and NH_4_^+^ in Aqueous Solution

**Figure 5:**
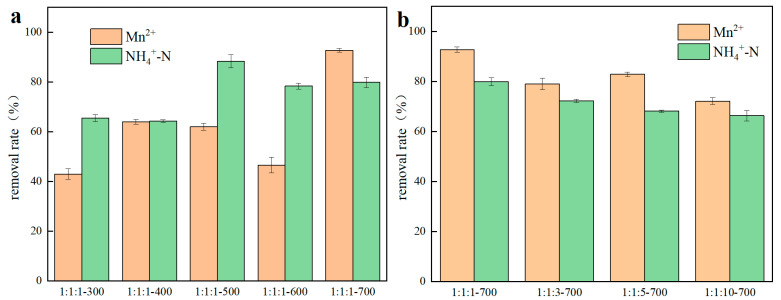

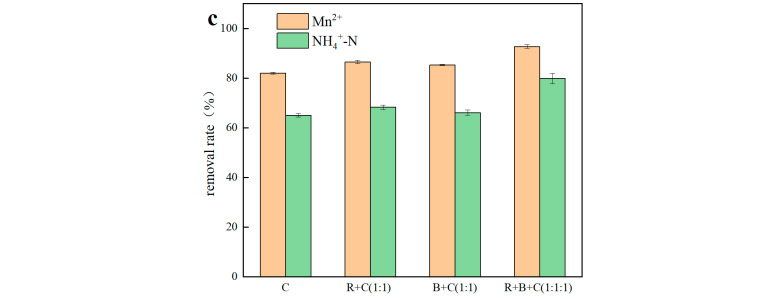
The removal effect of Mn^2+^ and NH_4_^+^-N (**a**) composite materials prepared at different decomposition temperatures (**b**) composite materials prepared at different ratios. (**c**) Single biochar and different composite materials.

The removal performances of Mn^2+^ and NH_4_^+^-N from solution by the composites synthesized at various pyrolysis temperatures (300–700 °C) with a constant precursor ratio (1:1:1 for red mud: bentonite: corn straw) are presented in Figure 5a. The results show that the removal efficiency of manganese by the composite material significantly increases with the rise of pyrolysis temperature. The material prepared at 700 °C has the highest removal efficiency for manganese, reaching 92.72% ± 0.85%. This enhancement is closely correlated with the increase in specific surface area and the more developed mesoporous structure at higher temperatures, which provide abundant active sites for Mn^2+^ adsorption and subsequent precipitation. Pearson correlation analysis revealed a strong positive correlation between BET surface area and Mn^2+^ removal efficiency (R^2^ = 0.94), whereas the correlation with NH_4_^+^ removal was weaker (R^2^ = 0.51), reflecting the dominant role of surface functional groups in NH_4_^+^ capture. Additionally, based on the XPS results, it is inferred that thermochemical reduction at 700 °C generates zero-valent iron (Fe^0^) species, which not only act as electron donors to create alkaline microenvironments but also facilitate the formation of stable Fe-Mn spinel phases via isomorphous substitution. However, the optimal removal rate of ammonia nitrogen (88.34% ± 1.2%) occurs under the pyrolysis condition of 500 °C. This observation can be attributed to the greater retention of oxygen-containing functional groups on the material surface at moderate pyrolysis temperature, as evidenced by the stronger FTIR absorption peaks at 3630 cm^−1^, 1500 cm^−1^, and 1026 cm^−1^ (Figure 3a). These polar groups are essential for NH_4_^+^ capture through electrostatic attraction and hydrogen bonding, outweighing the benefit of higher surface area at 700 °C for this specific pollutant. The slight decrease in Mn^2+^ removal efficiency at 600 °C (compared to 500 °C and 700 °C) may be attributed to the partial melting of the red mud-derived aluminosilicate glass phase at this temperature, which partially blocks pores and reduces the accessible surface area. This is consistent with the SEM observation (Figure 1d) showing the formation of a glassy phase at 600 °C. At 700 °C, further melting and re-crystallization may create a more stable pore structure, recovering and even enhancing the surface area.

Figure 5b shows the removal performance of the composite materials prepared at a fixed pyrolysis temperature (700 °C) and different raw material ratios (1:1:1, 1:1:3, 1:1:5, 1:1:10) for Mn^2+^ and NH_4_^+^-N. The results show that as the proportion of biomass added increases, the removal efficiency of both pollutants by the composite material shows a downward trend, indicating that an excessively high proportion of biomass is not conducive to the removal of manganese and ammonia nitrogen. When the raw material ratio is 1:1:1, the composite material exhibits the best removal performance, with the removal efficiency of manganese reaching 92.72% ± 0.85% and that of ammonia nitrogen reaching 79.87% ± 0.62%.

Figure 5c presents the control experiments conducted with materials prepared under identical conditions (700 °C, 2 h, N_2_ atmosphere) from single corn straw, binary mixtures (red mud + corn straw, bentonite + corn straw), and the ternary mixture (red mud + bentonite + corn straw), tested in Mn^2+^ and NH_4_^+^-N solutions. The results show that the ternary composite (1:1:1-700) exhibits significantly higher removal efficiency than any binary composite or pure biochar, confirming the synergistic effect among the three components.

**Figure 6:**
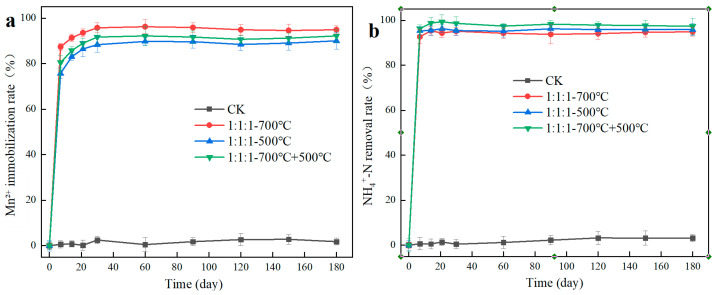
(**a**) Mn^2+^ immobilization rate (**b**) NH_4_^+^-N removal rate.

The results of the long-term soil column experiment are presented in Figure 6. All treatment groups amended with the composite exhibited significant immobilization effects on Mn^2+^ in the soil. Within the initial 7 days of the experiment, all three treatments achieved Mn^2+^ immobilization rates exceeding 75%, indicating the rapid responsiveness of the composites, which is primarily attributed to the abundant active sites on the material surface as well as rapid ion exchange and surface complexation reactions [33]. Among them, the group treated with only the composite prepared at 700 °C exhibited the highest immobilization rate (96.22% ± 0.5%), followed by the group treated with both composites prepared at 700 °C and 500 °C (92.17% ± 0.72%), and the group treated with only the composite prepared at 500 °C showed an immobilization rate of 90.03% ± 1.2%. This difference can be explained by the influence of pyrolysis temperature on the microstructure and surface chemical properties of the materials. The higher pyrolysis temperature (700 °C) promoted the graphitization of the biomass-derived carbon skeleton and the melting/reconstruction of iron- and aluminum-bearing minerals in the red mud, resulting in a larger specific surface area, a more developed micro-mesoporous structure, and a higher density of oxygen-containing functional groups (e.g., –OH, –COOH). These features enhanced the physical adsorption, surface complexation, and co-precipitation of Mn^2+^ [34,35]. In contrast, the material prepared at 500 °C exhibited incomplete carbonization, limited pore development, and lower active site density, leading to a slightly inferior immobilization capacity.

Regarding the removal efficiency of ammonia nitrogen, the experimental group with the combined addition of composites prepared at both temperatures exhibited the best performance (99.33% ± 0.23% removal), which was significantly superior to that of the single-material groups (96.33% ± 0.54% for the 500 °C group and 95.34% ± 0.82% for the 700 °C group). This phenomenon reveals a synergistic mechanism of composites derived from different pyrolysis temperatures: the higher temperature (700 °C) may partially reduce Fe_2_O_3_ in the bauxite residue to zero-valent iron (Fe^0^), which is inferred to act as an electron donor that facilitates the selective oxidation of NH_4_^+^ to N_2_ gas, as supported by the observed ORP decrease and literature evidence, while the well-developed porous structure of the 700 °C material facilitates the physical entrapment and diffusion of NH_4_^+^; meanwhile, the abundant polar functional groups (e.g., carboxyl and hydroxyl groups) retained on the surface of the 500 °C material efficiently capture NH_4_^+^ through electrostatic attraction and ion exchange [36]. The combined use of the two materials creates a cascading effect of “adsorption–catalytic oxidation–readsorption”. Although direct monitoring of Fe, Al, and other element release was not performed in this study, the significant reduction in Mn^2+^ and NH_4_^+^ leaching (as determined by HJ/T 299-2007) and the alkaline conditions maintained throughout the experiment suggest that the composite does not introduce additional leaching risks. Future studies should include systematic monitoring of major and trace element release during long-term application.

### 3.3. Remediation Performance of Composite Materials in Manganese Slag-Contaminated Soil

#### 3.3.1. Variations of Soil Properties Throughout the Remediation

**Figure 7:**
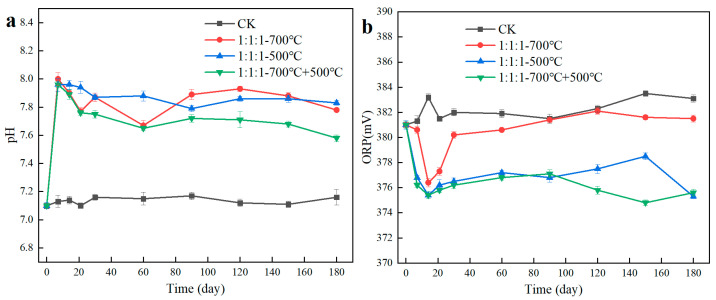
(**a**) the pH of the soil (**b**) the ORP of the soil.

During the 180-day remediation process, both soil pH and ORP underwent changes. As shown in Figure 7a, following the addition of the composite, the soil pH increased sharply from approximately 7.1 to 7.9–8.0 during the initial period (0–10 days), and then decreased slightly but remained alkaline overall. This is attributed to the release of OH^−^ and the rapid hydrolysis of alkaline minerals from the red mud, which significantly enhanced the soil alkalinity [37]. Under high-pH conditions, free Mn^2+^ tends to form insoluble manganese hydroxide or carbonate precipitates. The differences observed among the various thermal treatment methods may be related to the release rates and species of alkaline substances from the composite. As shown in Figure 7b, compared with the control group (CK), the soil ORP of all treatment groups decreased during the initial stage (to approximately 376 mV), with the 700 °C + 500 °C group maintaining the lowest ORP throughout the later stage, indicating the introduction of reductive components by the composite. This is attributed to the partial reduction of Fe_2_O_3_ in the red mud to zero-valent iron via high-temperature thermal reduction, which served as an electron donor and lowered the redox potential [38]. Combined with the high-pH environment, a synergistic mechanism of “reductive release and high-pH precipitation” was established: the low ORP environment may facilitate the reduction of Mn oxides (Mn^3+^/Mn^4+^) present in the soil to Mn^2+^, which is the mobile form of manganese. However, in this system, the released Mn^2+^ is immediately immobilized through precipitation and lattice incorporation under the alkaline conditions provided by the composite. Thus, the reduction of Mn oxides does not increase the environmental risk of Mn^2+^; rather, it promotes the conversion of all manganese species into more stable solid phases. Under alkaline conditions (pH7.9–8.0), a significant portion of NH_4_^+^ exists as NH_3_ (pKa = 9.25), which reduces the electrostatic attraction between NH_4_^+^ and the negatively charged surface of the composite. However, the porous structure of the composite still allows physical adsorption of NH_4_^+^/NH_3_. The low ORP environment is conducive to the Fenton-like reaction, as the presence of Fe^0^ and Fe^2+^ can generate reactive oxygen species (·OH) under aerobic conditions. The combined addition of 700 °C and 500 °C composites resulted in the lowest ORP because the 700 °C sample provides a sustained source of Fe^0^ (electron donor), while the 500 °C sample retains more oxygen-containing functional groups that may facilitate electron transfer. The synergistic effect of these two components creates a more reducing environment than either material alone.

**Figure 8:**
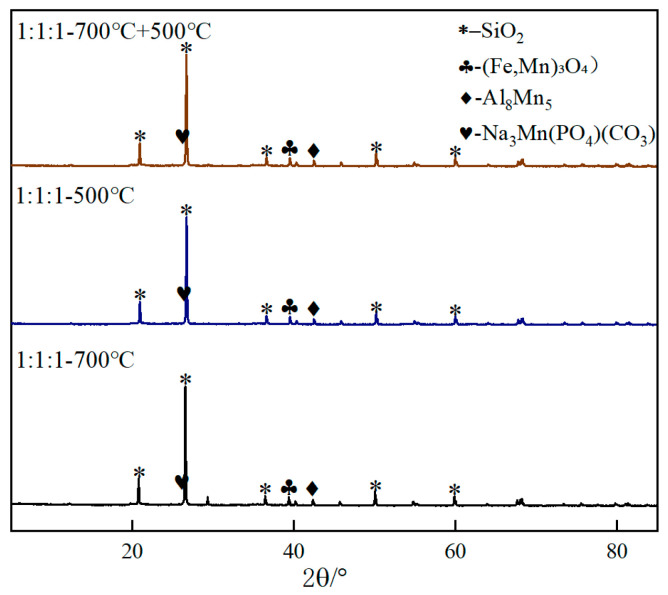
XRD of the remediated soil.

Based on the XRD pattern analysis, the mineral compositions of the soils after remediation in the three experimental groups (1:1:1-700 °C, 500 °C, and 700 °C + 500 °C) were highly consistent. The main detected phases included quartz (SiO_2_ PDF #46-1045), an iron-manganese oxide solid solution ((Fe,Mn)_3_O_4_ PDF #75-0033), an aluminum-manganese compound (Al_8_Mn_5_ PDF #42-0021), and a sodium manganese phosphate-carbonate composite salt (Na_3_Mn(PO_4_)(CO_3_) PDF #45-0567). No significant differences in the types of neoformed phases were observed among the experimental groups, indicating that the immobilization pathway of manganese is universal. The stable immobilization of manganese can be attributed to the dual mechanisms of mineral lattice solid solution and chemical precipitation. On the one hand, the iron components in the remediation material undergo isomorphous substitution with free Mn^2+^, forming a highly stable spinel structure (Fe,Mn)_3_O_4_, thereby achieving co-precipitation immobilization [39]. On the other hand, the high-pH environment provided by the red mud promotes the reaction of Mn^2+^ with phosphate and carbonate ions, generating an insoluble Na_3_Mn(PO_4_)(CO_3_) mineral phase. Regarding ammonia nitrogen, no nitrogen-containing crystalline phase was detected in the XRD patterns, indicating that ammonia nitrogen was not immobilized in the solid phase as mineral salts; its removal mainly relied on catalytic oxidation to N_2_ gas. It should be noted that the composite material was mixed with soil at a relatively low addition rate (3%), and physical separation of the composite from the soil matrix after 180 days of remediation was not feasible. Therefore, the XRD patterns presented in Figure 8 represent the bulk remediated soil (soil + composite system). Nevertheless, compared with the untreated soil (Figure S3), the appearance of new diffraction peaks corresponding to (Fe,Mn)_3_O_4_, Al_8_Mn_5_, and Na_3_Mn(PO_4_)(CO_3_) in the remediated soil provides evidence for Mn^2+^ immobilization through lattice incorporation and precipitation. However, the sustained remediation performance over 180 days (Figure 6) and the formation of stable secondary phases (Figure 8) suggest that the composite maintains its structural integrity and reactive functionality throughout the experiment.

**Figure 9:**
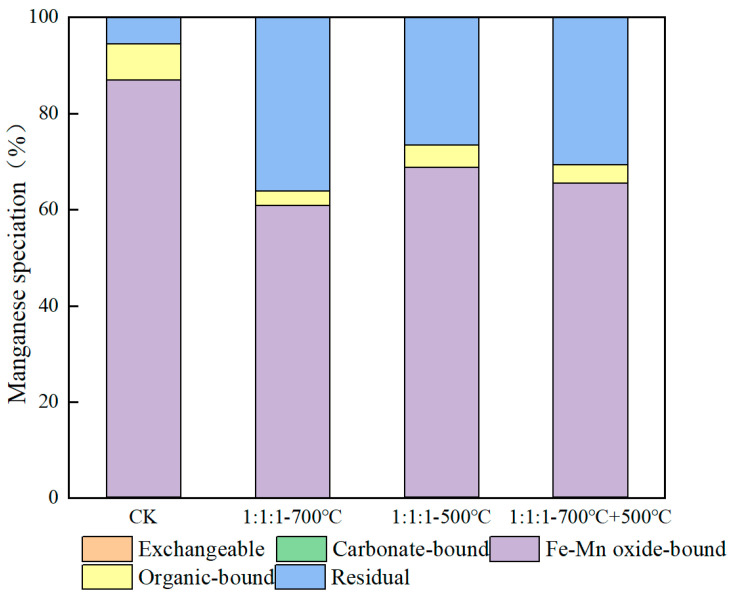
Manganese speciation in soil.

Based on the manganese speciation distribution shown in Figure 9, each remediation treatment significantly altered the manganese fractions compared with the control (CK). In the CK, manganese was predominantly present as the Fe-Mn oxide-bound fraction (85%), accompanied by minor amounts of the organic-bound and residual fractions. After the application of the red mud-based composites, the proportion of the most stable residual fraction increased significantly from approximately 5% in the CK to 25–35%, while the Fe-Mn oxide-bound fraction decreased relatively yet remained dominant (60–70%), and the organic-bound fraction decreased. A small amount of carbonate-bound fraction appeared in the 700 °C and composite-temperature groups. The potentially bioavailable fractions were at very low levels. The substantial increase in the residual fraction can be attributed to mineral lattice solid solution and chemical precipitation. Under the high-pH environment, free Mn^2+^ underwent isomorphous substitution with iron from the material, forming a highly stable (Fe,Mn)_3_O_4_ spinel phase. Meanwhile, Mn^2+^ reacted with phosphate and carbonate ions to generate insoluble minerals such as Na_3_Mn(PO_4_)(CO_3_), which were directly anchored into the crystal lattice. The decrease in the organic-bound fraction was due to the alkaline environment promoting the dissolution or decomposition of organic matter, leading to the release of complexed manganese, which then re-entered more stable pathways, i.e., binding to Fe-Mn oxides or being fixed into the mineral lattice [40]. In summary, the system achieves long-term efficient stabilization of manganese through the dual mechanisms described above. The formation of Fe–Mn spinel solid solution is suggested by the appearance of (Fe,Mn)_3_O_4_ diffraction peaks in the XRD patterns (Figure 8) and the substantial increase in the residual Mn fraction from 5% to 25–35% after remediation (Figure 9). The residual fraction is widely recognized as the most stable form, in which metals are incorporated into the crystal lattice of minerals [41]. However, specific desorption experiments under varying pH and redox conditions would be valuable for future work to further confirm long-term stability. These observations collectively imply the incorporation of Mn into spinel-type phases through isomorphous substitution. However, direct characterization such as TEM-EDS or high-resolution XPS Mn 2p analysis would be beneficial in future studies to provide more definitive evidence.

#### 3.3.2. Underlying Mechanisms

**Figure 10:**
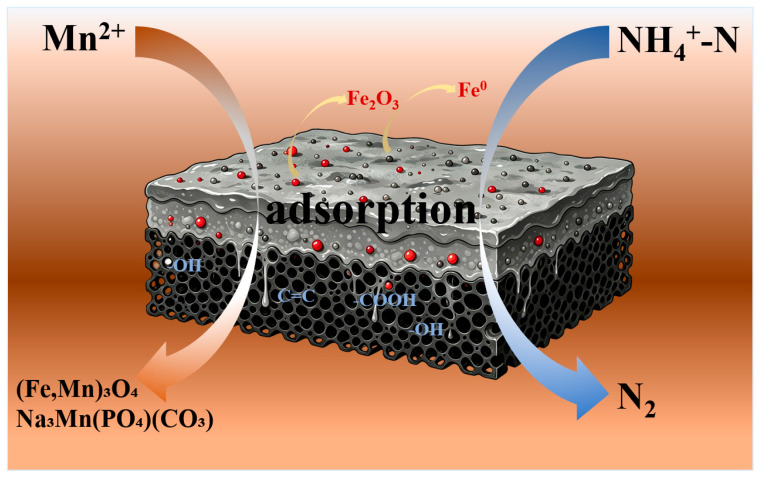
Proposed pathways for simultaneous Mn immobilization and NH_4_^+^-N removal by red mud-based porous carbon thermal composite material.

The composite, featuring abundant pore structures and the inferred presence of zero-valent iron (Fe^0^) particles as suggested by XRD and SEM-EDS analyses, achieve synergistic immobilization of Mn^2+^ and removal of NH_4_^+^-N in soil. As illustrated in Figure 10, Mn^2+^ is first enriched on the carbon surface via adsorption. During the microenvironmental corrosion of Fe^0^ (Reaction (1)), OH^−^ is generated, creating a locally high-pH micro-interface on the material [42]. This alkaline condition significantly promotes the transformation of Mn^2+^ into highly stable spinel phases ((Fe,Mn)_3_O_4_) and complex manganese phosphate-carbonate composite salts (Na_3_Mn(PO_4_)(CO_3_)), thereby achieving efficient and stable immobilization of manganese. For the removal of ammonia nitrogen (NH_4_^+^-N), the removal mechanism involves the synergistic effects of adsorption and oxidation. First, the composite possesses a well-developed pore structure and abundant surface functional groups, enabling effective capture and enrichment of NH_4_^+^ from the soil solution onto the material surface and within its pores via electrostatic attraction, ion exchange, and surface complexation, thus achieving preliminary solid–liquid separation. Subsequently, the inferred Fe^0^ on the porous carbon skeleton undergoes corrosion under aerobic conditions (Reaction (1)), generating in situ Fe^2+^ and H_2_O_2_, which are proposed to trigger a Fenton-like reaction (Reaction (2)) generating hydroxyl radicals (·OH) as the reactive species [43]. It should be noted that ·OH radicals are highly reactive transient species with an extremely short lifetime, making direct detection challenging, particularly in complex soil–composite systems. Therefore, the involvement of ·OH is inferred from indirect evidence, including the selective oxidation of NH_4_^+^ to N_2_ (without significant accumulation of NO_3_^−^ or NO_2_^−^) and the Fe^0^-driven ORP decrease (Figure 7b), as well as literature support [44,45,46]. The enriched and adsorbed NH_4_^+^ is then attacked by the proposed ·OH (Reactions (3) and (4)), undergoing a radical chain reaction to be completely oxidized to harmless N_2_ gas. This process forms a closed loop of “adsorption–enrichment–oxidative degradation”: adsorption not only reduces the concentration of NH_4_^+^ in the solution but also provides a high-concentration reaction interface for the subsequent oxidation, thereby significantly enhancing the removal efficiency and mineralization degree of ammonia nitrogen.

2Fe^0^ + O_2_ + 2H_2_O → 2Fe^2+^ + H_2_O_2_ + 2OH^−^ (1)

Fe^2+^ + H_2_O_2_ → Fe^3+^ + ⋅OH + OH^−^ (2)

NH_4_^+^ + ⋅OH → ⋅NH_2_ + H_2_O + H^+^ (3)

2NH_4_^+^ + 3⋅OH → N_2_↑ + 3H_2_O + 4H^+^ (4)

## 4. Conclusions

This study aimed to develop an efficient, low-cost functional material from solid waste (red mud, bentonite, and corn straw) for the simultaneous remediation of Mn^2+^ and NH_4_^+^ in manganese slag-contaminated soil. The main conclusions are as follows:

- Pyrolysis temperature significantly influenced the material properties: higher temperatures (700 °C) promoted carbonization and pore development, while moderate temperatures (500 °C) retained more oxygen-containing functional groups. The optimal mass ratio was 1:1:1 (red mud: bentonite: corn straw), which balanced pore structure development and active site density.
- In the 180-day soil column experiment, the addition of this composite significantly reduced the leaching toxicity of Mn^2+^ and NH_4_^+^ in manganese residue-contaminated soil. Among the treatments, the material prepared at 700 °C achieved a Mn^2+^ immobilization rate as high as 96.22% ± 0.5%, while the mixed addition of materials prepared at 700 °C and 500 °C exhibited the best removal efficiency for NH_4_^+^, reaching 99.33% ± 0.23%.
- The composite achieves efficient and stable stabilization of manganese through a multi-mechanism process involving adsorption, precipitation, and lattice solid solution. The alkaline environment provided by the material promotes the transformation of Mn^2+^ into insoluble carbonates, while the oxidation products of Fe^0^ form a stable spinel phase (e.g., (Fe,Mn)_3_O_4_) with Mn^2+^, anchoring manganese into the mineral lattice.
- The removal of ammonia nitrogen is proposed to result from the synergistic effect of adsorption and catalytic oxidation. The porous structure adsorbs and enriches NH_4_^+^, while Fe^0^ is inferred to generate hydroxyl radicals (·OH) via a Fenton-like reaction, which are proposed to oxidize NH_4_^+^ to N_2_ gas, thereby forming a closed loop of “adsorption–enrichment–oxidative degradation”.
- Continuous monitoring over 180 days showed that the remediation effect increased with time, indicating that the composite possesses good long-term stability and sustained remediation capability, making it suitable for in situ remediation of manganese residue-contaminated sites.

## Figures and Tables

**Table 1:** Elemental analysis of the surface of composite materials.

Composite Materials	Elementary Composition (%)				Atomic Ratio		
	C	N	O	H	H/C	O/C	(O + N)/C
1:1:1-300	11.09	0.37	19.33	1.62	0.15	1.74	1.78
1:1:1-400	14.05	0.3	16.58	0.89	0.06	1.18	1.20
1:1:1-500	13.54	0.32	14.60	1.19	0.09	1.08	1.10
1:1:1-600	14.63	0.43	12.43	1.63	0.11	0.85	0.88
1:1:1-700	17.32	0.49	13.53	0.55	0.03	0.78	0.81
1:1:3-700	21.55	0.37	10.46	0.92	0.04	0.49	0.50
1:1:5-700	23.08	0.4	10.93	0.94	0.04	0.47	0.49
1:1:10-700	35.47	0.55	11.42	1.27	0.04	0.32	0.34

**Table 2:** Specific surface area of composite materials.

Composite Materials	Specific Surface Area (m^2^/g)	Pore Volume (cm^3^/g)	Average Pore Diameter (nm)	Pore Size Distribution
1:1:1-300	28.38	0.066	24.69	Mesopore-dominated
1:1:1-400	30.26	0.073	20.78	Mesopore-dominated
1:1:1-500	50.87	0.081	13.32	Mesopore-dominated
1:1:1-600	55.69	0.089	11.65	Mesopore-dominated
1:1:1-700	74.27	0.092	7.88	Mesopore-dominated
1:1:3-700	57.79	0.098	9.39	Mesopore-dominated
1:1:5-700	61.54	0.095	9.00	Mesopore-dominated
1:1:10-700	43.07	0.078	9.37	Mesopore-dominated

## Data Availability

The original contributions presented in this study are included in the article/Supplementary Material. Further inquiries can be directed to the corresponding author.

## Supplementary Materials

The following supporting information can be downloaded at: https://www.mdpi.com/article/10.3390/ma19143076/s1.
