# Supplementary material for: Waste Control by Waste: Red Mud-Based Porous Carbothermal Composite for Efficient Remediation of Manganese and Ammonia Nitrogen in Contaminated Soil
Source: Materials (Basel). 2026 Jul 17;19(14):3076. doi: 10.3390/ma19143076 (PMC13414390; doi:10.3390/ma19143076)
Supplement: Supplementary file 1 [file materials-19-03076-s001.zip › materials-4360884-supplementary.pdf]

Supporting Information

Waste Control by Waste: Red Mud-Based Porous Carbothermal Composite for Efficient Remediation of Manganese and Ammonia Nitrogen in Contaminated Soil

Xinyue Shi [1,2,3,4], He Shang [1,2,3,4], Lei Wang [1,2,3,4,*], Hongxia Li [1,2,3,4], Meilin Liu [1,2,3,4] and Yingchun Sun [1,2,3,4,5]

[1] National Engineering Research Center for Environment-Friendly Metallurgy in Producing Premium Non-Ferrous Metals, China GRINM Group Co., Ltd., Beijing 101407, China; shixinyue@grinm.com (X.S.); shanghe@grinm.com (H.S.); lihongxia@grinm.com (H.L.); liumeilin@grinm.com (M.L.); 15966001661@163.com (Y.S.)

[2] GRINM Resources and Environment Tech. Co., Ltd., Beijing 101407, China

[3] National Engineering Research Center for Environment-Friendly Metallurgy in Producing Premium Non-Ferrous Metals, Beijing 101407, China

[4] Beijing Engineering Research Center of Strategic Nonferrous Metals Green Manufacturing Technology, China GRINM Group Co., Ltd., Beijing 101407, China

[5] General Research Institute for Nonferrous Metals, Beijing 100088, China

* Correspondence: wanglei@grinm.com; Tel.: +86-15801002031

This supporting information contains:

number of pages: 3

number of tables: 4

number of figures: 3

Table S1. Chemical composition of red mud.

element	Fe_2O_3	CaO	Al_2O_3	SiO_2	TiO_2	Na_2O	ZrO_2	MnO	MgO	Cr_2O_3	SO_3
content (wt%)	37.44	19.21	14.66	12.11	8.39	6.55	0.33	0.24	0.23	0.21	0.21

Table S2. Chemical composition of bentonite.

element	SiO_2	Al_2O_3	Fe_2O_3	MgO	CaO	Na_2O	K_2O	TiO_2	P_2O_5
content (wt%)	51.16	17.49	6.48	1.40	4.12	1.39	1.08	0.76	0.23

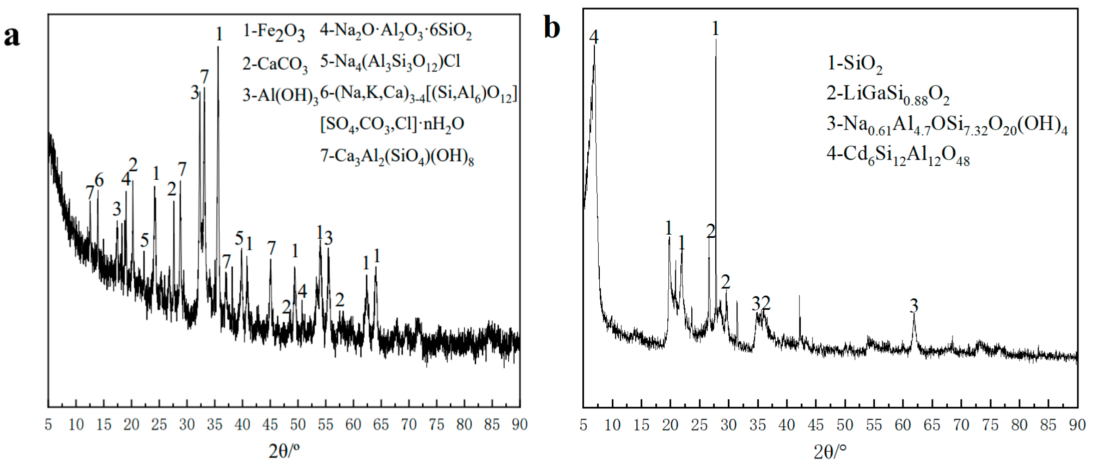

Figure S1. (a) XRD of red mud. (b) XRD of

bentonite **Table S3.** Elemental analysis of the biochar.

composite materials	elementary composition（%）				atomic ratio		
	C	N	O	H	H/C	O/C	(O+N)/C
Y-300	37.85	1.59	20.20	3.40	0.09	0.53	0.58
Y-400	44.13	1.41	15.09	2.96	0.07	0.34	0.37
Y-500	46.52	1.35	13.53	2.22	0.05	0.29	0.32
Y-600	47.40	0.91	12.63	1.46	0.03	0.27	0.29
Y-700	48.9	0.86	12.38	1.53	0.03	0.25	0.27

Table S4. Adsorption capacity of composite materials

composite materials	Mn^{2+} Removal (%)	q_e, Mn^{2+} (mg/g)	NH_4^+-N Removal (%)	q_e, NH_4^+-N (mg/g)
1:1:1-300	42.98±2.14	85.96±4.28	65.54±1.45	32.77±0.73
1:1:1-400	64.05±0.98	128.1±1.96	64.27±0.56	32.14±0.28
1:1:1-500	62.03±1.45	124.06±2.9	88.34±2.67	44.17±1.34
1:1:1-600	56.6±3.16	113.2±6.32	78.37±1.17	39.19±0.59

1:1:1-700	92.72±0.85	185.44±1.7	79.87±0.62	39.94±0.31
1:1:3-700	78.95±1.09	157.9±2.18	72.27±0.67	36.14±0.34
1:1:5-700	82.87±0.87	165.74±1.74	68.17±0.45	34.09±0.23
1:1:10-700	72.16±1.34	144.32±2.68	66.32±2.15	33.16±1.08

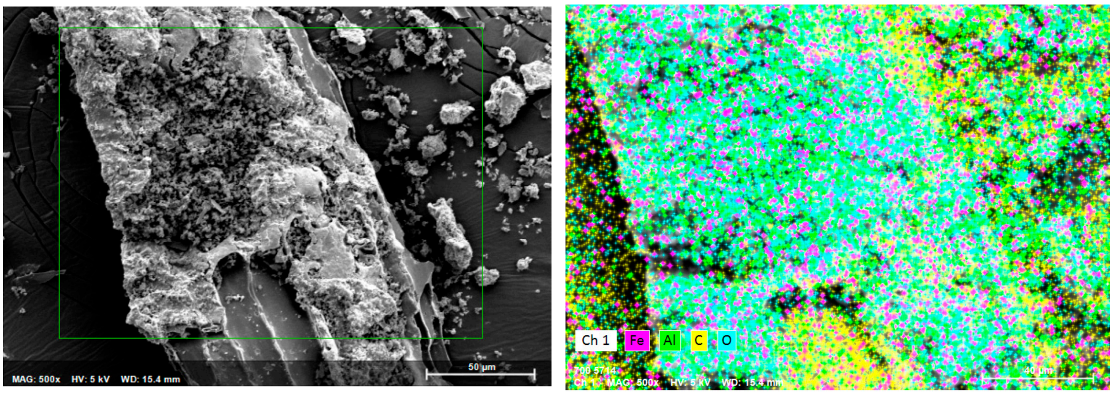

Figure S2. SEM-EDS images of the composite materials（1:1:1-700）

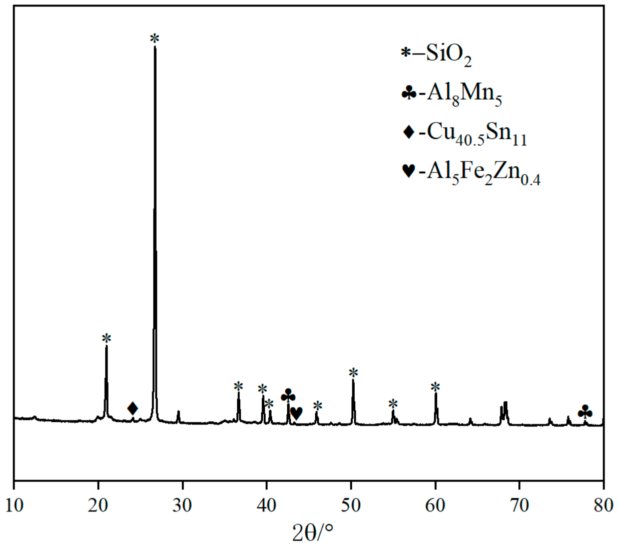

Figure S3. XRD of the untreated soil
